# Cholera, Snake-bite and Their Lessons

**Published:** 1884-10

**Authors:** P. P. Wells

**Affiliations:** Brooklyn


### HOMŒOPATHIC PHYSICIAN.

A MONTHLY JOURNAL OF MEDICAL SCIENCE.

"If our school ever gives up the strict inductive method of Hahnemann, we are lost, and deserve only to be mentioned as a caricature in the history of medicine."—CONSTANTINE HERING.

Vol. IV. OCTOBER, 1884. No. 10.

### CHOLERA, SNAKE-BITE AND THEIR LESSONS.

P. P. WELLS, M. D., BROOKLYN.

There are dangers and calamities to which mankind is exposed, of different degrees of destructive power, before some of which our nature shrinks instinctively, and, judging from the ordinary experience of them, it would seem that defense or deliverance from them are to be sought in the Divine power alone. Educated science as met among men can only stand before them appalled and confess its powerlessness to give relief. Among these are malignant cholera and the bite of the rattlesnake. Cholera continues to seize its victims and quench their lives even before the eyes of the "*cholera experts*," including Koch, just the same since he proclaimed his discovery of the germs he alleges to be the originating cause of the plague as before his discovery, and before its history or its presence to-day self-styled "*scientific medicines*" stands as to its cure precisely in the attitude of Faust:

> " *Da steh ich nun, ich armer thor,*

> *Und bin so klug als wie zufore!*"*

To cure or to prevent, after the opportunities of half a century for observation and study of this opprobrium, "scientific medicine" is as powerless in the end as in the beginning. It has learned nothing. It has not even learned that it has learned nothing. To be sure, it claims to have discovered the "*cholera microbe.*" But what then?

* *Vide* HOMŒOPATHIC PHYSICIAN, Vol. IV, p. 30.

Why, destroy these—*tolle causam*—and lo! the resulting effects must cease. All that's needed is a "*germicide*," and then the problem is solved. So it would seem. But the sad fact is, after much great and earnest "*stamping out*" of these causing "microbes," cholera continues and kills just the same as before. And this is all "scientific medicine" has found for our protection and healing when before this dread plague. Why is this? Simply because it has been looking in a wrong direction for a solution of this problem. It has been seeking it under the guidance of worthless theories and traditions, and accepting only what was believed to be in harmony with these; and they have not found it simply because it was not there. They were in the position of the mariner who looked due *west* for the *pole star*. The end of such navigation and such so-called medical observation can both only end in disaster.

We have said "scientific medicine" has learned nothing in this half century of opportunity as to prevention or cure of this saddest and most fatal of plagues. The noxious nature of filth was known before. It would seem to have *forgotten* some of the conclusions of its own earlier experiences, if it has learned nothing by subsequent observations. In the epidemic which started in Northeastern Hindostan in 1828 or 1829,* and reached Western Europe in 1831 and the United States in 1832, the "scientific medicine" of that day learned that the drug on which its theories had caused it chiefly to rely for its successful treatment was not useful at all as a remedy, but only and at all times *hurtful*, and the more if its use were combined, as their theory suggested it should be, with brandy. The drug was opium. The one only thing learned in that epidemic was that neither opium nor brandy was of the least value, whatever theory might have suggested as to their probable utility in treating malignant cholera. This one point, then learned, it would seem, has been forgotten, for at least one government has reduced its tariff of duty on imported opium, in an expected great need and demand for the drug in treating this expected disease. This policy can hardly have been otherwise suggested than by "scien-

*This epidemic started in the *northeast* of Hindostan and crossed the peninsula to its *southwest* border against the southwest monsoon, a wind which blows with force night and day, without intermission, during its period of continuance. This could not have happened if its originating cause had been minute material organisms. Minute material bodies could, in these circumstances, only move in the direction of the wind current. The fact that this epidemic moved *only* in the exact opposite direction, effectually disposes of Koch's microbes as its cause, and negatives his claim for them of this character, beyond all possible reply.

tific medicine." It is from this source governments take their inspiration in matters of sanitary policy and action.* And yet this same authority found out, more than fifty years ago, that this drug was not only useless but positively hurtful in the treatment of cholera patients. "Scientific medicine" of to-day seems to have forgotten this, its one only discovery of half a century ago, and opium is to be made cheaper that its uselessness and harmfulness may be more largely and apparently displayed. We have known something of the blinding power of "scientific" conceit, heretofore, but this seems to be its latest if not its clearest demonstration.

In a retrospect of the history of "scientific medicine," other and many sicknesses and accidents are seen before which the experience and confession of poor, dazed Faust is paralleled, and to-day before these it is as empty of all knowledge of ways and means of cure as it was centuries ago. Of these it may be said, as of cholera, it has "learned nothing." Why? Not from lack of opportunity for observation and study of these ofttimes fatal sicknesses or accidents. Not from any want of acuteness of intellect in the ranks of the representatives of "scientific medicine." Not for any lack of zeal or industry on their part. Not because the powerlessness for cure in these ranks was not recognized and acknowledged. Then why, in all these years, is the sad fact justly charged against them as to these opprobria. "They have learned nothing"? The obvious answer to this inquiry is, Because they have groped for the knowledge they sought in absolute darkness, or only in such light as speculations and baseless theories can give, and these, as to any beneficial results, have been but total darkness. They would neither accept better methods nor a better light, and the sad result is and has been, "*learned nothing*"! They have sought for hints as to ways and means of cure in post-mortem dissections with a diligence worthy of much admiration, but in these there has been and is no voice of instruction as to means of cure of that which had caused the discovered changes in tissues or organs; and yet, wonderful it is, these votaries of "scientific medicine" have not in all these centuries even learned this silence of morbid anatomy as to all belonging to a discovery of

---

*The same is true also of those newspaper men who often attempt to enlighten the public on matters of health and cures. One of these, a correspondent of one of our daily papers of largest circulation, informs his readers that "opium and laudanum are the *only* remedies for cholera"! He, unquestionably, had been so informed by the doctors, who had only forgotten what their predecessors had learned in their so great and fatal experiences of these drugs in 1828–1832.

curing agents. After any fatal termination of a new pathological puzzle they rush to the dissecting table and the scalpel for light which shall give them a better understanding of the problem if, perchance, it may be met again, and perhaps with hopes of finding some fact which may throw light on means for cure. In these many thousands of examinations they have never once found such fact, and is it not curious that now, after centuries of such examinations and such failures, the rush is still as prompt and earnest to the table and the knife, and the search there is just as honest and diligent for that which, in the nature of things, can never there be found, as it was in the beginning of these voiceless examinations so many centuries ago, just as if the unvarying experience of the past in these had not been an unvarying disappointment? Is it not curious? In this, as in the general question of ways and means of cure, the verdict is and must be, "*They have learned nothing;*" the true explanation of which is, "*Ye will not come to the light!*"

The newest exhibition of imbecility of this "scientific" practice before grave accident which we have seen is found in the history of the treatment of the last snake-bite. As before cholera, so here, powerless for good is the conclusion all intelligent minds must accept as the outcome of the endeavors for the relief of the bitten man. Alcohol and Ammonia, and then death, this being all "scientific medicine" had to offer for prevention of this result. And more, it was declared this, though a failure, was "all that was known" of means of relief from the effects of this poisoning. This was all the opportunity for observation and study of this kind of poisoning through the centuries of the past history of "scientific medicine" has gathered of knowledge of means for its cure—a poverty of results so remarkable that the rest of mankind may well marvel at it, and, perhaps not unreasonably, question the character of the claimed "*scientific*" element of the dominant practice of "*medicine.*" *Snake-bite*, Alcohol and Ammonia, and then death, and this is declared by the medical attendant to be "*all that was known*" of curatives for snake-bites—that is, this was all he knew, and before those interested in the fate of this poisoned man he was pretending to possess all of existing knowledge on this subject in possession of any man. This was either his mistake or his crime—his mistake if he believed his statement true; his crime if he knew better. In the great multitude of curing agents known more or less perfectly to those whose work it is to acquaint themselves with the powers of these agents, there are many which may be selected as curatives of the phenomena of such poisonings, as these, in their progress, may develop different facts, the cure depending on the selection of the right agent, as demanded by law, for the group of phenomena present at the time of its administration.

In contrast with this imbecility, as before the cholera problem, should be placed the history of Hahnemann's dealing with this problem when the epidemic of 1831 was creeping toward the eastern border of Europe. When fear of this great plague which had left the dead in so great numbers along the track of its approach had thrown the whole population into agitation and alarm, this great man alone was tranquil and calm, confident of his mastery of this dread enemy. He told the doctors and the people what the medicines were which would both cure and prevent cholera;* and subsequent experience of the use of these drugs abundantly proved the truth of his predictions. These drugs *did* both cure and protect the people from attack. How did he find this secret? What did he do? As he had then never seen a case of cholera, there could have been no microscopic hunting for microbes.† This has been reserved for the

* "Scientific medicine," having learned nothing as to a successful treatment of cholera, has continued its groping, and groping in darkness, for methods and means of cure in this latest European outbreak of the malady the same as at the first, notwithstanding this method of Hahnemann, proclaimed at the approach of the first epidemic into Europe, proved both curative and prophylactic, as he had predicted; and notwithstanding the truth of this prediction has been so abundantly confirmed in the experiences of several subsequent epidemics, both European and American, "scientific medicine" continues to ignore this experience, treats the many-thousand-times-demonstrated truth of Hahnemann's prediction, and the fact of the enormously greater success of his method of dealing with cholera as compared with its own, with affected scorn and contempt. Does it, in this, pretend to have found a better method? Nay, it has found none at all. It has only groped in darkness, and in the darkness of its theories, for that sought-for light, and, in a word, with these has resorted to no small experimentation, all of which, a physician in the Pharo Hospital writes to a correspondent of the New York *Times*, have proved "*failures.*" Such experiments have always proved failures. And now, after these so many failures, are these "scientific" gentlemen any more ready to heed the better experiences of those who have cured and protected by use of Hahnemann's method? Not in the least. Their own failures, on the one side, and the comparatively superb successes, on the other, are not regarded by them. If these successes are mentioned to them they only imitate the pride and scorn of the Pharisees of old, who received a report from the man who was blind, with the proud rebuke, "Dost *thou* teach *us?*" The hospital physician above referred to characterizes the people of Marseilles thus—and the language and character can be no less appropriate if applied to "scientific" medical gentlemen in general, before this cholera problem—"The ignorance and bigotry of the population [doctors] here are almost incredible."

† These microbes, Koch hastily and boldly declares, are the true cause of malignant cholera. He just as positively declares that their life, and therefore their noxious activity, depends on moisture; that if once *dry* they are *dead.* This is his second *fact,* so called, discovered and proclaimed beside his amusement of "scientific medicine" of a later day. And yet, let it be remembered, with no thought of "microbes," he found the cure. He had previously found the way to this, and the way was through God's law of relationship of curatives to the sicknesses to which He had appointed them for their healing. As in contrast with our more modern searches by our modern "scientific medicine," we see God's law on the one side and the microscope on the other, and by the law the cure and prophylactic were found, while the microscope only found microbes. Surely this difference of result should be accepted by all mankind as conclusive of the superiority of law over all speculations, however ingenious, as a guide in searching out the secret of curatives for the sick.

This result should be instructive to all doctors and all mankind;—to doctors, teaching them the fact of the existence of this law and its absolute authority in all duties and processes of healing, and to the rest of mankind that all pretense of a "scientific" character of any therapeutic endeavor outside of this law is only a sham and a *false* pretense. The result of the search under law should have been equal to quenching the hate of even "scientific medicine" for all pertaining to this law; but history shows that it has only intensified this, and the more whenever law is found opposing its speculations, however baseless these may really be. Is it not these which give to it its only claim to the title so dearly cherished by it—"scientific"? Then, "out with law, since either this must go or our pride!"

So before the problem of a cure for snake-bite. It may well be conceived that a search for its solution under the guidance of law might have resulted in the discovery of other remedies than these speculative ones, Alcohol and Ammonia, and these others may have wrought a cure. *Certainly* this would have been the first—the existence of these microbes. The facts in the case of cholera prove that even an earnest, honest, and industrious man may be mistaken. If *dead* when dry, and because dead powerless to produce the disease, is a fact, then the other fact—that cholera was produced by exposure to clothing removed from a box where it had been closely packed eight years, which clothing eight years before had been charged with the cholera atmosphere—proves the existence of some other cause of the disease than Koch's microbes. Koch has only discovered their existence; not that they are in any way important in causation, prevention, or cure. If the causing agent in this case were microbes, and these only cause cholera when moist, they were certainly a long time in drying and dying. The drying and dying may safely be stamped with the verdict which intelligence of the facts of cholera will surely impress on this whole microbe discovery as a cause not only "not proven," but *false*. All Koch has discovered is the presence of these organisms in the excreta of cholera patients. His ascription to them of the office of causation is wholly gratuitous.

result if the *specific* for the case had been found; and this could only have been found by a search under and according to law. If found and given to the poisoned man there might then and there have been demonstrated the difference in the results of *specific* and "*scientific*" medicine.

The reason why "scientific medicine" has no remedies for snake-poisoning is because it has no guides in its search for them other than its unscientific speculations, and these have been proved wholly inadequate to the work. It has despised God's law as a guide, and so each has become a blind guide to himself, and the centuries of search under this direction has ended in "no other remedies known than Alcohol and Ammonia." And, as it was pretty well known that the last snake-bite treated in New York had vast administration of Alcohol, and yet the patient died, it might not unreasonably be questioned by some whether this be really a remedy for this formidable poisoning. Indeed, so great was the quantity given that some, perhaps not unreasonably, questioned whether Alcohol had not *killed* the patient. It certainly did not cure him. And, as in this case, the "scientific" rule for drug administration—"all he can bear"—was very faithfully and fully carried out, it cannot be denied that the *killing* was a possibility. Was the *killing* any better by reason of the claim that it was "scientific"?

Then, in this last poisoning, after death, it is curious to see how, in strict accord with "scientific" tradition, the rush was to the table and the knife—and for what? To find in the dead hints as to any possible cure of the living while so poisoned? Then they were certainly looking for what no similar examinations had ever yet disclosed in any similar or any other cases. The facts which point to the specific curative for any case are found in the living, never in the dead. In the case of serpent-poisonings these facts are to be sought and found, if found at all, just as specific curatives are sought and found in *all* other sicknesses, *i. e.*, under the guidance of law, and not otherwise. Law requires that the agent which can produce in the living organism phenomena the most like those of the sick condition shall be found and given as its corollaries require, and then it promises us a cure. We know of no reason for believing serpent-poisonings are any exceptions to this relationship of curatives to sicknesses which so many thousands of experiences in other forms of sickness have so fully demonstrated and confirmed. Find the agent which has been known to have produced phenomena most like those resulting from this poisoning, and the law says you have its cure. Are any such agents known? Let us see, if we may, how our priceless Lachesis corresponds in its poisonous results to those of the Crotalus. The symptoms of Crotalus here given are from the *Encyclopœdia of Materia Medica.* Those of Lachesis are, except those credited to Jahr, from his *Codex*, and those followed by B. from Bœnninghausen's "*Taschen Buch*," and those by H. from Hering's "*Schlangen Gift.*"

| CROTALUS. | LACHESIS. |
|---|---|
| Depression of spirits and indifference. | Depression of spirits, with chilliness. Indifferent and disinclined to work. |
| Delirium at night. | Delirium.—B. |
| Dullness of intellect. | Mentally very indolent. |
| Can't express his ideas. | |
| Weakness of memory. | Weakness of memory, so it was difficult to remember what was said to him. |
| Vertigo. | Vertigo (chronic after the bite). |
| Vertigo, with nausea. | Vertigo previous to the vomiting. |
| Vertigo, with paleness. | Vertigo precedes faintness. |
| Headache extending to the eyes. | Pressure above left eye. Violent throbbing above left eye. Pressure over eyes extending to root of nose. |
| Pressive frontal headache. | Pressure in the forehead. |
| Pains in temples. | Headache in both temples. |
| Pressure in temples, especially the left. | Pressure quite severe in left temple. |
| Stitches in left temple or whole left side of the head. | Stitches in left temple and side of the head. |
| Itching of scalp. | Violent itching of scalp, as if from ants. |
| Yellow color of eyes. | Yellowness of the sclerotica.—*Jahr.* |
| Frequently of the whole body. | Jaundice.—B. H. |
| Blood exudes from the eyes. | Bleeding from eyes.—*Jahr.* |
| Blood flows from eyes, ears, and nose. | Bleeding from eyes, ears, and nose.—*Jahr.* |
| Pains above the eyes. | Pains above the eyes after weeping. |
| Eyes almost closed. | Swelling gradually extended over the whole face, so that eyes became closed. |
| Frequent lachrymation. | Eyes watery, with nasal catarrh. |
| Bleeding from the ears. | Bleeding from the ears.—*Jahr.* |
| Stoppage of the ears. | Singing in right ear, which afterward felt as if closed. |
| Bleeding from nose. | Bleeding of the nose. |
| Earthy color of the face. | Earthy, gray color of the face. |
| Yellow color of face. | Yellow color of the face during the fever. |
| Leaden color of face. | Leaden color of face.—*Jahr.* |
| Cramps in jaw. | Spasms of the jaw.—*Jahr.* |
| Bleeding of the gums. | Bleeding of the gum. |
| Swelling of the tongue, with inflammation of it. | Left half of the tongue swollen, feeling as though it were the effects of mercury. |

| | |
|---|---|
| Accumulation of saliva. | Salivation, with biting on tongue. |
| Articulation indistinct. | Speech indistinct.—*Jahr*. |
| Loss of speech. | Difficult speech.—*Jahr*. |
| The tongue and whole throat as if constricted. | Pressure in the throat, as if from something astringent. |
| Dryness of the throat, with great thirst. | Evenings and nights the throat as if dried up with thirst. |
| Painful rawness of the throat. | Rawness of the throat, with swelling. |
| Larynx painful to touch. | The throat very sensitive to external pressure. |
| Great thirst from the first. | Constant thirst, with dry tongue and skin. |
| Unquenchable, burning thirst. | Insatiable thirst, with dry mouth and weakness. |
| Nausea, with vertigo and headache. | Nausea, then vomiting, with transient vertigo. |
| Nausea and vomiting soon after the bite. | Nausea, with vomiting and thirst. |
| Constant vomiting. | Constant inclination to vomit. |
| Vomiting of food. | Vomiting of food. |
| Vomiting of blood. | Vomiting of blood. |
| Intolerance of clothing about the epigastrium. | He was obliged to loosen his clothes. |
| Painfulness of the epigastrium. | Pain in epigastrium while pressed upon. |
| Stitching in the epigastrium. | Stitching extending from the stomach to the chest. |
| Stomach sore and tender. | |
| Throbbing in the epigastrium. | Throbbing in the epigastrium. |
| Violent, burning umbilical pains. | Burning sensation in the umbilicus. |
| Swelling of the whole abdomen. | Abdomen distended hard. |
| Burning pains in the abdomen, with great sensitiveness to touch. | Burning in the abdomen, with unendurable pain and sensitiveness to touch. |
| Bleeding from anus. | Discharge of blood and pus from anus. |
| Urine reddish yellow. | Urine dark yellow, color of copper coin. |
| Sexual excitement during the day. | Unusual erections during the day. |
| Impotence. | Continued absence of sexual power. |
| Voice weak, hoarse, and rough. | Voice hoarse, with a rough throat. |
| Spitting of blood. | Expectoration of blood or of blood and frothy mucus. |
| Respiration difficult and slow. | Respiration difficult almost all day. |
| Oppressed respiration while sitting. | Unable to breathe, must sit up. |
| Pain in the breast. | Spasmodic pains, which cause anxiety and palpitation. |
| Pulse full and rapid—then full and slow. | Pulse full and hard. |
| | Pulse small and rapid, with hot skin. |
| Pulse weak and intermitting. | Pulse small, weak, and irregular. |
| Pulse tremulous. | Pulse small, soft, and irregular. |
| Whole extremities swollen and very painful. | The bitten limbs become inflamed and swollen. |
| Heaviness in the arms and legs. | Heaviness of all the limbs. |
| Swelling and heaviness of the bitten arm. | Aching and heaviness extending through shoulder and arm. |
| Cold swelling of hand and arm, with painfulness to pressure. | Swelling of the muscles of the forearm, frequent swelling of the hand. |

| | |
|---|---|
| Trembling of hands. | Trembling of the hand in a drunkard.—*Cured.* |
| Swelling and burning pain in the leg that was bitten. | Gangrene of the bitten part, with inflammation and swelling of the part. |
| Flesh decayed and fell from the bitten leg. | The flesh mortifies and falls off in pieces.—H. |
| Œdematous swelling of the whole body. | Œdema.—B. |
| Hæmorrhage from all the orifices of the body. | Bleeding from eyes, ears, nose, anus, urethra.—*Jahr* and H. |
| At times the blood flows suddenly from eyes, ears, nose, gums, and beneath the nails. | |
| General spasms, without foaming at the mouth. | Convulsions and other spasms, with violent outcries. |
| Depression of vital powers. | Great exhaustion of body and mind. |
| Great loss of muscular power. The least exertion tires. | Great relaxation of muscles and exhaustion from the least exertion. |
| Tremulous weakness. | Weakness, especially of the legs and arms. |
| Paralysis of one side. | One-sided paralysis, especially of the arms. |
| Faintness. | Frequent faintness. |
| Faintness, with imperceptible pulse and inclination to vomit. | Frequent faintings, with nausea, dispnœa, palpitation of heart, and cold sweat. |
| All the pains alternate rapidly with each other and frequently recur. | Pains pass from one part to another, first from left then from right. |
| Yellow color of the whole body. | Jaundice.—B. and H. |
| Yellow spots over the body. | Yellow spots. |
| Oozing of blood in the form of sweat in large quantities. | Bloody sweat.—B. |
| Gangrene of the bitten portion, extending over the body. | The bitten wound swells, is ecchymosed, the red becomes black, bluish, red-spotted, black-spotted, yellow-spotted, insensible, and gangrenous. —H. |
| Great sleepiness. | Great sleepiness.—H. |
| Coma. | Coma.—H. |
| Many dreams of strife and anger. | Laborious dreams. |

These symptoms have been taken from the record as those most likely to be met in cases one may be called to treat, as they are, most of them, such as speedily follow the bite. The similarity of the two series is certainly very great, and it would seem, if similarity is the constituent of the curative in all cases, that here in Lachesis we have a cure for poisonings by the Crotalus which can leave little need of any other. The similarity here is much greater than that of either Alcohol or Ammonia. But just here we are met by a caution from the father of Lachesis himself. In *Archiv. fur Homœopathische Heilkunst*, b. xv, *theil* 1, s. 13, he says: "Many experiences have made plain to me the law, 'Near relationships of earths, plants, or animals are, notwithstanding all relationship of their symptoms (*i. e.*, the similarity of them), by no means mutual antidotes to each other.' " He illustrates this by instancing Rhus poisoning not antidoted by other varieties of Rhus;* nor Nux vomica by Ignatia; Solanum dulcamara by Solanum nigrum; nor Veratrum by Sabadilla. This is true. And he says of the Trigonocephalus Lachesis that it is "only a great rattlesnake without the rattles," *i. e.*, the poisons of the two are too near *identity* to fulfill the office of similarity. He certainly is not wise who lightly regards any practical experience of Hering, or any proclamation by him of a law pertaining to practical therapeutics. He says the antidote should be sought in agencies far removed in scientific relationship from that which has caused the evil to be cured.

If a high potence of Lachesis should disappoint us, we should naturally next resort to that most similar animal poison which after Lachesis has shown nearest relationship to the phenomena of the case. The poison of some of the deadly insects of the tropics would very naturally suggest itself—of the spiders, for example—though, perhaps, the similarity of the experience of poisonings by these is more in their rapidly fatal terminations than in the morbid phenomena they present—Hering's Theridion, for example. There is also a spider in our own climate the bite of which is followed very speedily by great pains and swelling of the parts bitten, and sometimes by rapidly fatal results. Then we should not forget Apis and Cantharis. Apis has the severe pain, rapid and extreme swelling, restlessness, anxiety, and rapid exhaustion of life-power and oppressed breathing, all so characteristic of crotalus poisoning. Cantharis, inflammation, swelling and vessication of the affected parts with great burning, all which are found in so great degree in these poisonings. Arsenic has likeness also in the extreme anxiety, restlessness, oppressed respiration, embarrassment of the heart's

---

*The best cure we know for Rhus poisoning is a *very high potence* of Rhus itself. I have seen obstinate chronic eruptions and ulcers from this poison disappear as by a charm on the patient receiving the 40,000 of Fincke, when lower potencies given in the same case had been wholly inoperative, as had also other remedies which in their results have symptoms similar to some of those of Rhus. Shall we be instructed by this fact, and in case of poisoning by the crotalus give Lachesis very high, with hopes of success from change of action of the crude Lachesis poison by the process of potentization? In the case of the Rhus the 10,000 and the 40,000 have cured cases for me when the 30, 12, 3, and even lower potencies have utterly failed to give any relief, proving a *different* action of these higher—the *idem* by this process seeming to have become a *simile*. We should certainly resort to a high potence of this most similar poison with good hopes of success.

action, and great exhaustion. Hering mentions Belladonna and Phosphorus as remedies of great value in treating crotalus poisoning. Their similar relationship to some of the phenomena of these poisonings is sufficiently apparent. The faintness with *slow* and *feeble* beating of the heart would also suggest as a relief of this grave fact, so threatening of the approaching paralysis of this organ now so certainly near and so certainly fatal if not speedily relieved, resort to *Laurocerasus*, which in its poisonings presents a very similar condition of heart function, though nothing like to that most fearful of all in the snake-poisoning —the tendency to rapid dissolution of all the fluids and solids of the organism. The Laurocerasus can, therefore, hardly be regarded as a curative of the whole train of phenomena of the poisoning, while it may be a most valuable agent for the arrest of this approaching paralysis, so speedily and certainly fatal if not promptly met and overcome by appropriate means. By this resort we may gain time in which we may be able to deal with other grave elements of the problem of the complete cure.

It can hardly be otherwise than that he who is guided in his selection of remedies by the light of law will find in the medicines named, or among the multitude of others whose record we have in our materia medica, many remedies for this fearful emergency far superior to the poverty-stricken, confessed limitation of resources of the old school, which only knows Alcohol and Ammonia. We say remedies far superior by reason of their greater similarity of action to that of the snake-poison. Intelligent men can hardly fail of being instructed by this comparison of the resources of the two schools, before this and other extreme emergencies, as to two facts: First, as to the greater number of agents for their cure in possession of the new, and the unspeakable advantage the physician has who seeks among our many medicines the one specific for his case, under the guidance of law; and second, the utter emptiness of the pretense of those who are, by their own confession, limited in their resources to the *two* agents so often named, and so recently proved to be powerless for relief, that theirs is the "*scientific*" medicine, and that as to all of this the world has they are its sole possessors and monopolists. It can hardly be otherwise than that in the practical application of the therapeutics of the two schools for the relief of this great danger, the great superiority of that governed and guided by law, over that whose only resource is guessing, will be demonstrated. Conceit, arrogance, and pride, in the long run, will be found but feeble enemies of law and orderly administration of means it demands and provides for the cure of the great plagues we have considered, as well as all other grave sicknesses to which men are liable.

